# Human-facilitated metapopulation dynamics in an emerging pest species, *Cimex lectularius*

**DOI:** 10.1111/mec.12673

**Published:** 2014-02-17

**Authors:** TOBY FOUNTAIN, LUDOVIC DUVAUX, GAVIN HORSBURGH, KLAUS REINHARDT, ROGER K BUTLIN

**Affiliations:** * Department of Animal and Plant Sciences, University of SheffieldWestern Bank, Sheffield, S10 2TN, UK; † Department of Biosciences, University of HelsinkiPO Box 65 (Viikinkaari 1), FI-00014, Helsinki, Finland; ‡ Institute for Evolution and Ecology, University of TübingenAuf der Morgenstelle 28, D-72076, Tübingen, Germany

**Keywords:** approximate Bayesian computation analysis, *Cimex lectularius*, genetic structure, metapopulation dynamics, microsatellites, pest management

## Abstract

The number and demographic history of colonists can have dramatic consequences for the way in which genetic diversity is distributed and maintained in a metapopulation. The bed bug (*Cimex lectularius*) is a re-emerging pest species whose close association with humans has led to frequent local extinction and colonization, that is, to metapopulation dynamics. Pest control limits the lifespan of subpopulations, causing frequent local extinctions, and human-facilitated dispersal allows the colonization of empty patches. Founder events often result in drastic reductions in diversity and an increased influence of genetic drift. Coupled with restricted migration, this can lead to rapid population differentiation. We therefore predicted strong population structuring. Here, using 21 newly characterized microsatellite markers and approximate Bayesian computation (ABC), we investigate simplified versions of two classical models of metapopulation dynamics, in a coalescent framework, to estimate the number and genetic composition of founders in the common bed bug. We found very limited diversity within infestations but high degrees of structuring across the city of London, with extreme levels of genetic differentiation between infestations (*F*_ST_ = 0.59). ABC results suggest a common origin of all founders of a given subpopulation and that the numbers of colonists were low, implying that even a single mated female is enough to found a new infestation successfully. These patterns of colonization are close to the predictions of the propagule pool model, where all founders originate from the same parental infestation. These results show that aspects of metapopulation dynamics can be captured in simple models and provide insights that are valuable for the future targeted control of bed bug infestations.

## Introduction

With increased travel and connectivity, there are now many opportunities for the human-facilitated dispersal of organisms, with disease vectors and pests of economic importance presenting a particular concern (Estoup *et al*. 2004; Grapputo *et al*. 2005; Tatem *et al*. 2006; Niggemann *et al*. 2009; Lawson Handley *et al*. 2011). Passive dispersal, coupled with high population turnover, can lead to organisms existing as highly structured metapopulations (De Meester *et al*. 2002; Haag *et al*. 2005; Walser & Haag 2012) with discrete breeding groups, frequent and independent local extinctions, and potential for patches to be recolonized (see Hanski 1999).

The population dynamics of metapopulations greatly affect how genetic diversity is distributed, both within and between local populations (Slatkin 1977; Wade & McCauley 1988; Hastings & Harrison 1994). Founder events can shift populations out of equilibrium by reducing genetic diversity and increasing genetic drift. Coupled with restricted migration, this can lead to rapid genetic differentiation of subpopulations (Wade & McCauley 1988; Whitlock & McCauley 1990). Frequent local extinction events can make differentiation even more extreme by limiting the ability of gene flow to equalize allele frequencies (Whitlock & McCauley 1990; Hastings & Harrison 1994; Pannell & Charlesworth 1999; Ray 2001).

Theoretical studies predict that the number and origin of colonists of each subpopulation have a strong influence on the degree of subpopulation differentiation. When colonists of any given subpopulation have originated from a unique parental population (propagule pool model), differentiation between subpopulations is always predicted to increase relative to the equilibrium with no extinction. This is in tandem with a reduction in neutral genetic variation both within subpopulations and throughout the entire metapopulation (Wade & McCauley 1988; Pannell & Charlesworth 1999). In contrast, a mixed origin of colonists (migrant pool model) may lead to an increase or decrease in differentiation (Wade & McCauley 1988). The numbers of founders and their relatedness to each other therefore directly influence the genetic variance between subpopulations, with kin-structured colonization and subsequent inbreeding leading to substantial differentiation (Whitlock 1992; Ingvarsson & Olsson 1997; Ingvarsson 1998; Johannesen & Lubin 1999; Torimaru *et al*. 2007).

Metapopulation dynamics are not only important for understanding many evolutionary processes but also have practical implications for conservation (Ray 2001; Frankham *et al*. 2002), control of invasive species (Lawson Handley *et al*. 2011) and integrated pest management (Collins *et al*. 2000; Rinkevich *et al*. 2007; Yakob *et al*. 2008). For example, selection for resistance alleles could reduce the chance of a subpopulation going extinct after a pest control treatment, thus increasing the period during which it acts as a source of migrants. This would facilitate the spread of resistance alleles into new subpopulations and severely hamper control. Knowledge of the determinants of population structure would help predict the spread of resistance alleles (Churcher *et al*. 2008).

The common bed bug (*Cimex lectularius*) is re-emerging as a significant economic and public health pest, precipitated by a sudden global resurgence in its populations (Boase 2001; Doggett *et al*. 2004; Kilpinen *et al*. 2008; Potter *et al*. 2008; Richards *et al*. 2009). The causes of this sudden population expansion have remained a mystery whose resolution has been hampered by a lack of research on the bed bug's basic population and dispersal biology (Reinhardt & Siva-Jothy 2007). Bed bugs are flightless, obligate blood-sucking insects that can form infestations comprising up to thousands of individuals (Reinhardt *et al*. 2010; Wang *et al*. 2010). Infestations typically consist of aggregations of individuals located in discrete refugia (e.g. a crack in a bed frame). Bed bugs walk to the host and return to a refuge when feeding is complete (Reinhardt & Siva-Jothy 2007). Being flightless, individuals can only move actively over limited distances, and much of their recent spread is attributed to long-distance passive dispersal facilitated by human movement (Doggett *et al*. 2004; Reinhardt & Siva-Jothy 2007; Potter *et al*. 2008; Szalanski *et al*. 2008).

In a bed bug metapopulation, human dwellings form habitat patches and it is expected, based on observations of bed bug dispersal, that small numbers of individuals found new infestations. Bed bugs have been observed actively dispersing throughout buildings (Doggett & Russell 2008; Wang *et al*. 2010), and it is likely that this not only accounts for propagation of infestations within buildings but is also the mechanism by which individuals move into portable items, leading to passive dispersal. Research on the composition of founders has been limited, and recent studies have given contrasting results. For example, Szalanski *et al*. (2008) found up to six mitochondrial haplotypes within a single infestation and Booth *et al*. (2012) showed evidence of multiple introductions within an apartment complex. These results seem consistent with a migrant pool model of colonization, indicative of a genetically diverse group of founders. In contrast, other infestations have been shown to have very limited within-infestation diversity (Booth *et al*. 2012; Saenz *et al*. 2012), which more closely follows the predictions of a propagule pool model. Local extinctions are especially frequent as infested properties are often treated with insecticides, giving the majority of occupied patches a relatively short lifespan. Reports of widespread insecticide resistance (Doggett *et al*. 2004; Romero *et al*. 2007; Potter *et al*. 2008) suggest that selection is further shaping the genetic diversity of bed bug metapopulations. In combination, these factors predict low diversity within and very high levels of structuring between subpopulations of bed bugs.

Despite its importance in the maintenance of genetic diversity, there has been limited research on the number and genetic composition of colonists in natural metapopulations (Gaggiotti *et al*. 2004 but see Whitlock 1992; Austin *et al*. 2011). Here, we provide detailed insight into bed bug population dynamics across two hierarchical levels of structure. First, we use classical population genetics approaches to characterize within-infestation and city-wide genetic diversity. Second, we simplify two classical models of metapopulation dynamics in a coalescent framework in order to evaluate their relative fit to the bed bug system as well as to estimate model parameters, particularly the number of colonists, using ABC. Therefore, this is the first study to use a model-based approach, with an explicit statistical framework, to test two alternative hypotheses on the genetic composition of founders in a natural bed bug metapopulation. We discuss the implications of these results for the future integrated control of bed bugs.

## Materials and methods

### Sampling and estimation of genetic diversity

#### Sample collection

To investigate within-infestation diversity, individuals were sampled from multiple refugia in each of five properties. These properties are subsequently referred to as AUS (sourced from New South Wales, Australia), BIR1 and BIR2 (separate properties located within Birmingham, UK), and LON1 and LON2 (separate properties from London, UK; Table S1, Supporting information). Detailed spatial surveys of properties LON1 and LON2 (including refuge location) are described in Naylor (2012), case studies 4 and 3, respectively. These infestations were selected from available samples on the basis of having individuals sampled separately from multiple refugia, rather than based on their geographical location.

To assess diversity at the city scale, 13 infestations from across London, UK, were sampled, pooling individuals from multiple refugia within each infestation (see Fig. S1, Supporting information, Table3 for names and spatial locations). Pest control operatives, who obtained individuals prior to the treatment of affected properties, provided the majority of samples. Once received, all samples were stored in screw-topped rubber-sealed microfuge tubes containing 1.5 mL of absolute ethanol (analytical reagent grade) at room temperature.

#### DNA extraction and genotyping

The ammonium acetate precipitation method described by Nicholls *et al*. (2000) was used to extract DNA. Individuals were then genotyped using 21 newly isolated microsatellite markers. The microsatellite loci were isolated from either a microsatellite-enriched genomic library or a recently available transcriptome assembly (O. Otti & K. Reinhardt, in preparation; see Supporting Information for a description of microsatellite isolation and characterization). All 21 loci used were confirmed as autosomal by the observed presence of heterozygotes in male and female individuals. The same PCR conditions were used as described for primer testing (see Supporting Information), with individuals genotyped at 19 autosomal loci for the within-infestation study and at 21 autosomal loci for the city-wide study. Amplified products were analysed using an ABI3730 48-well capillary DNA analyser, and allele sizes were assigned using GENEMAPPER v.3.7 (Applied Biosystems). Sequences were searched against the NCBI nonredundant nucleotide database using BLASTn, which confirmed that these markers did not overlap with previously published bed bug microsatellites (Booth *et al*. 2012).

#### Genetic diversity

For each data set, descriptive summary statistics including number of alleles and expected (*H*_E_) and observed (*H*_O_) heterozygosities were obtained using Microsatellite Analyser version 4.05 (Dieringer & Schlötterer 2003). Allelic richness was calculated using *F*_STAT_ version 2.9.3.2 (Goudet 1995). We tested for deviations from Hardy–Weinberg equilibrium (HWE) and estimated the frequency of null alleles with cervus version 3.0.3 (Kalinowski *et al*. 2007). Evidence of linkage disequilibrium was assessed using genepop version 4.1.0 (Raymond & Rousset 1995; Rousset 2008). For analyses of deviation from HWE and evidence of linkage disequilibrium, a Bonferroni correction was applied to account for multiple testing (Rice 1989).

#### Population structure

We used *F*_STAT_ to calculate global *F*_IS_ and *F*_ST_ values (Weir & Cockerham 1984), both within and among infestations. Values were jackknifed over loci to give means and standard errors and bootstrapped over loci to give 95% confidence intervals. Ten thousand permutations were used to generate significance values.

The within-city data set was tested for isolation by distance amongst individuals in spagedi version 1.3 (Hardy & Vekemans 2002) using the kinship coefficient (Loiselle *et al*. 1995). Distance was partitioned into 10 intervals, with a uniform number of pairwise comparisons per interval. The mean distance value of each interval was log-transformed (Rousset 1997). We used 10 000 permutations to test whether the slope of the relationship between geographical and genetic distance was significantly negative.

For both data sets, we performed a discriminant analysis of principal components (DAPC; Jombart *et al*. 2010) using the adegenet 1.3–4 (Jombart 2008) package in R (R Core Team 2012) to examine evidence for genetic clusters, using infestation as a grouping prior. DAPC is an ideal clustering method for this data set as it does not make some commonly required assumptions (e.g. HWE; Jombart *et al*. 2010), which are unlikely to hold for bed bug infestations. The first step in DAPC is to transform the raw data into principal components (PCs). There is a trade-off in the number of retained PCs, with a higher number of PCs increasing the ability to discriminate between groups at the cost of the reduced stability of membership probabilities (Jombart *et al*. 2010). We used *a*-score as a measure for judging the optimal number of retained principal components. The *a*-score is the difference between the proportions of successful observed discriminations and values obtained from random discrimination. This was calculated with 100 permutations for each increasing number of retained principal components using the *optim.a.score* function in adegenet. Due to the low number of sampled individuals in each group, we were conservative with the number of retained principal components (PCs), but for both data sets, the number of retained PCs still incorporated ≥75% of the variance in the data. The *dapc* function was then used to perform the clustering analysis, and results are presented as ordination plots.

### Approximate Bayesian computation analysis

Approximate Bayesian computation allows inference without explicit calculation of likelihoods in complex scenarios with many parameters. It compares summary statistics from observed data to summary statistics from data simulated using various prior distributions (Beaumont 2010). In population genetics, where it is becoming a widely used tool, ABC analysis often involves the construction of models of population history, simulation of many data sets (e.g. using a coalescent approach) and comparison of simulated to observed data using summary statistics describing genetic diversity. This provides a statistical framework to compare different demographic models and then infer demographic parameters of interest [for a review of the global ABC procedure see Csilléry *et al*. (2010)].

#### Modelling rationale and implementation

Our aim was to construct simple models that nevertheless allow us to capture what is known of bed bug biology and to retain the key differences between competing hypotheses about colonization dynamics. By keeping the models as simple as possible, they can be assessed with the data available and relevant parameters can be estimated. We note that an important aspect of the bed bug system is the severe bottlenecks experienced by local populations during establishment of new infestations (Doggett *et al*. 2004; Saenz *et al*. 2012). These successive bottlenecks lead to the loss of most information concerning ancient demographic events, and inferences are thus restricted to the few most recent colonizations. We therefore choose to contrast simplified versions of the ‘propagule pool’ and ‘migrant pool’ models in a coalescent framework rather than trying to simulate colonization, migration and extinction more fully in a classical metapopulation framework. Our models still capture the main difference between these two metapopulation models, and their simplicity allows easier parameter estimation (see below). We used ABC to test the posterior probabilities of the two hypotheses and to estimate parameters for the preferred scenario. To demonstrate the wide applicability of this approach, we conducted all ABC analyses with the readily available and user-friendly software package diyabc (version 1.0.4.46) (Cornuet *et al*. 2008).

#### Model specification

**Figure 1:**
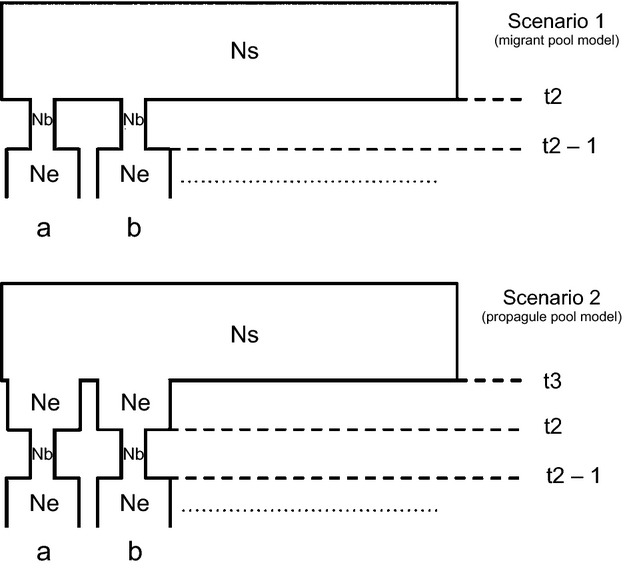
Demographic scenarios for ABC. *Scenario one: Migrant pool model of colonization*: All populations originate from a single hypothetic source population (Ns), which represents the metapopulation as a whole. At time t2, these populations diverge signifying the founding of new infestations, which includes a severe bottleneck (Nb). After a bottleneck of one generation, the populations grow and reach effective population size (Ne). It is this size at which the infestations are sampled. *Scenario two: Propagule pool model of colonization*: in this scenario, founders diverge from Ns at t3 and maintain a population size of Ne until t2 where there is a founding event and a severe bottleneck (Nb) of one generation. The subpopulation then grows to a size of Ne before being sampled. In both scenarios, only infestations a and b are shown, but models incorporate 13 sampled infestations, represented by the dotted line.

The two scenarios used in our ABC analysis are illustrated in Fig. 1. For both, we simulated 20 microsatellites from 13 infestations using the same sample size per infestation as obtained from the field (Table3). Note that from our 21 original microsatellites, the locus Cle001 was omitted, due to a large range of allele sizes that reduced our ability to fit the models. For the migrant pool model, we approximated the multiple origins of new infestations by randomly drawing individuals from a large panmictic population serving as a proxy for the metapopulation (scenario one). The propagule pool model was designed such that colonists founding a particular infestation all came from the same source infestation, itself derived from the large panmictic population (scenario two; Fig. 1). In both cases, we would expect a severe bottleneck at the point of founding, followed by a period of rapid growth. Because infestations are believed to be invariably settled through strong bottlenecks and to have short lifespans, we do not expect them to differ widely in age at sampling time or in effective population size. We thus simplified the models by constraining Ne, t2 and t3 to be shared by all infestations.

#### Prior distributions and summary statistics

To specify informative priors, we used results from case studies of infestations. Across 83 sampled infestations, there was a range from eight to approximately 100 000 individuals (How & Lee 2010; Wang *et al*. 2010; Naylor 2012), with a geometric mean of 93 individuals. We therefore initially selected a prior range for the average effective size of an infestation at the time of sampling (Ne) bounded between 10 and 3000 with a loguniform distribution. However, initial model runs suggested that this was an overestimate so we reduced the prior range to a loguniform distribution bounded between 10 and 100 (Table 1). Based on observed female fecundity, infestations can reach over 3000 individuals in as little as two to six generations. Due to this rapid growth, we fixed the bottleneck at one generation and selected a loguniform prior for time of founding (t2) bounded between two and 10 generations. Previous studies and our own data have suggested that infestations are started with small numbers of founders (Doggett *et al*. 2004; Saenz *et al*. 2012); we therefore set a uniform prior for this parameter (Nb) bounded between two and 14 individuals. This captures the range of possibilities from a single, once-mated founding female through a single, multiply mated female to a larger group of individuals. Population growth was simulated as a stepwise increase from Nb to Ne at time t2–1. For parameters where data were not available, we used a two-step approach starting with wide priors and then using narrower ranges that still captured whole posterior distributions (Table 1). As there is no existing knowledge on the mutation rate of short tandem repeats in bed bugs, we made the assumption that microsatellite loci followed a generalized stepwise-mutation model (Estoup *et al*. 2002). This model is defined by two parameters: μ, the mean mutation rate across loci, and *P*, the parameter of the geometric distribution describing the number of repeat changes per mutation event. As implemented in diyabc, each locus has its own μ_*i*_ and *P*_*i*_ drawn from a gamma distribution of mean μ and *P*, respectively, with shape parameter set to *k* = 2. All loci had a possible range of 40 contiguous allelic states, and we used diyabc's default prior ranges for mean μ and *P* (Table 1). We expected global diversity to depend on the product of the source population size, Ns, and the mutation rate μ, because the vast majority of mutations must have occurred before the first modelled colonization events. We did not expect to be able to estimate Ns and μ independently, but this does not impact our ability to estimate the parameters describing recent colonization and expansion events.

**Table 1:** Details of prior and posterior distributions of model parameters. Parameters constrained such that Ns > Ne > Nb

Parameters	Prior range	Mean	Median	Mode	HPD90 low	HPD90 high	RMAE
Ns	Loguniform [100–50 000]	6320	4950	2460	1520	15 200	0.416
Ne	Loguniform [10–100]	33.5	32.6	35.6	12.7	57.1	0.258
Nb	Uniform [2–14]	6.21	5.27	3.00	2.09	13.1	0.345
t2	Loguniform [2–10]	3.97	3.13	2.00	1.75	9.36	0.456
t3	Uniform [11–100]	52.2	49.6	26.8	18.3	94.1	0.266
Mean μ	Loguniform [10^−4^–10^−3^]	3.02 × 10^−4^	2.22 × 10^−4^	1.00 × 10^−4^	1.07 × 10^−4^	7.86 × 10^−4^	0.382
Mean P	Uniform [0.1–0.3]	0.115	0.103	0.100	0.100	0.167	0.243

RMAE computed using 500 pseudo-observed data sets taking the medians of posterior distributions as point estimates.

RMAE, relative median of the absolute error.

The type and number of summary statistics used in ABC is important to the analysis (Beaumont 2010). Although recent methods exist to choose or define the best possible summary statistics (Wegmann *et al*. 2010; Aeschbacher *et al*. 2012), no such approach is implemented in diyabc. We therefore selected, from among summary statistics available in the program, those shown to be informative in previous population genetic ABC studies (Cornuet *et al*. 2008; Lombaert *et al*. 2011; Dutech *et al*. 2012). These were mean number of alleles, mean genic diversity (Nei 1978) and mean allele-size variance for each population and pairwise population comparisons of *F*_ST_ (Weir & Cockerham 1984), giving a total of 39 single sample statistics and 78 pairwise comparisons. Because our strategy to choose summary statistics was not shown to be optimal, we pragmatically chose to use goodness-of-fit (GoF) analyses to check the robustness of our results (see Parameter estimation and model checking).

#### Simulation and model posterior probabilities

We simulated 1 × 10^6^ genetic data sets for each of the two scenarios. As an initial check that these scenarios could simulate data sets close to our observed data, we performed principal components analysis (PCA) on the summary statistics of the first 100 000 simulated data sets and evaluated the position of our observed data. To estimate the posterior probabilities of both scenarios, a polychotomous weighted logistic regression was performed on the closest 1% of simulated data to the observed data (Cornuet *et al*. 2008). Confidence in scenario choice was calculated by estimating false discovery rate (FDR) using 500 pseudo-observed data sets (POD; Cornuet *et al*. 2010; Duvaux *et al*. 2011). The basic idea is to generate PODs under the scenario with the lower posterior probability (PP) and to perform model choice using each POD in turn, in place of the real data. The FDR is then estimated by recording how many times we observe a PP equal or superior to the real PP of the best model.

#### Parameter estimation and model checking

Assessing the GoF of the chosen model is a critical step in model-based approaches (Gelman *et al*. 1995). We therefore ran posterior predictive simulations by creating 500 PODs from the posteriors of the chosen model. A PCA was then performed on summary statistics from (i) these 500 PODs, (ii) 500 data sets of each model randomly obtained from their priors and (iii) our observed data (Cornuet *et al*. 2010). As in Cornuet *et al*. (2010), we used a different set of summary statistics to perform the model checking compared with those used to calculate the posterior distributions of parameters in order to avoid overestimation of fit. We used Garza-Williamson's M (Garza & Williamson 2001) as a single sample statistic, and mean genic diversity and classification index (Rannala & Mountain 1997) between populations. The probability that the observed and the simulated data were significantly different was calculated by ranking the observed value of each test statistic against those obtained from the simulated data. *P*-values were corrected for multiple comparisons (Benjamini & Hochberg 1995).

To evaluate the posterior distribution of parameters, we performed a local linear regression on the closest 1% of logit-transformed simulated data. To assess confidence in our parameter estimates, we performed ABC analyses on 500 PODs created using values drawn from our prior distribution. Differences in the ABC point estimates and the POD true values were used to compute the relative median of the absolute error (RMAE – Cornuet *et al*. 2010).

## Results

### Characterizing *Cimex lectularius* infestations

In total, 21 microsatellite markers were found to be polymorphic and were assembled into five ABI four-dye multiplex sets using the program Multiplex Manager (Holleley & Geerts 2009; Table S2, Supporting information). To assess within-infestation genetic structure, 154 samples were genotyped from within five infestations at 19 of these loci. At the level of the whole infestation, only LON2 showed a significant departure from HWE after Bonferroni correction. Overall, low genetic diversity was detected within infestations with a high number of monomorphic loci, low allelic richness and low observed heterozygosity (Table 2). An *F*_IS_ value of −0.216 suggested an excess of heterozygotes within the AUS infestation (Table 2), which may be a signature of a recent bottleneck (Cornuet & Luikart 1996). Overall, estimates of *F*_IS_ were variable between infestations. This variability probably also reflects small numbers of founders. Note that the estimates are based on variable numbers of loci because of the occurrence of monomorphic loci within each infestation, consistent with low overall diversity, especially within BIR1, BIR2, LON1 and LON2. Within infestations, significant differentiation between refugia was only observed within LON2 (*F*_ST_ = 0.144, *P* = 0.008; Table 2). Five of 55 pairwise *F*_ST_ comparisons between LON2 refugia were significant, and no refuge was significantly differentiated from more than 27% of the other refugia (Table S3, Supporting information). In contrast, pairwise *F*_ST_ comparisons between infestations ranged from 0.492 to 0.834 (Table S4, Supporting information) and DAPC (Fig. S2, Supporting information) showed very high levels of differentiation between infestations.

**Table 2:** Genetic diversity and structure within five *Cimex lectularius* infestations for which multiple refugia were sampled. Total is the value obtained when individuals for all localities were pooled together. Heterozygosity and F-statistics were calculated within and among *C. lectularius* infestations at 19 loci. Significance of *F*_ST_ values was calculated after 10 000 permutations

Infestation	*n*	Allele range	Allelic richness† (±SE, n=17)	*L*_m_	*H*_E_	*H*_o_	*F*_IS_ (95% CI)	*F*_ST_
AUS	41	1–4	1.71 (0.15)	3	0.280	0.334	−0.216 (−0.349, −0.035)	0.017^NS^
BIR1	8	1–3	1.61 (0.12)	7	0.173	0.054	0.727 (0.476, 0.926)	−0.184^NS^
BIR2	9	1–5	2.10 (0.24)	6	0.380	0.337	0.141 (−0.104, 0.394)	−0.044^NS^
LON1	52	1–3	1.19 (0.09)	13	0.075	0.084	−0.135 (−0.232, −0.013)	0.010^NS^
LON2	46	1–3	1.62 (0.16)	8	0.250	0.163	0.219 (0.079, 0.475)	0.144*
Total	156	1–5	2.98 (0.15)	7.4	0.566	0.194	0.052 (−0.072, 0.198)	0.709**

Two loci were omitted because they failed to amplify for any individual in one infestation.

*n*, Number of individuals; *L*_M_, Number of monomorphic loci; *H*_E_, Expected heterozygosity; *H*_o_, Observed heterozygosity.

† Sample sizes standardized to the smallest number of individuals for a locus.

NS, nonsignificant (*P* > 0.05)

* significant (*P* < 0.01)

** highly significant (*P* < 0.001).

### City-wide genetic diversity

**Figure 2:**
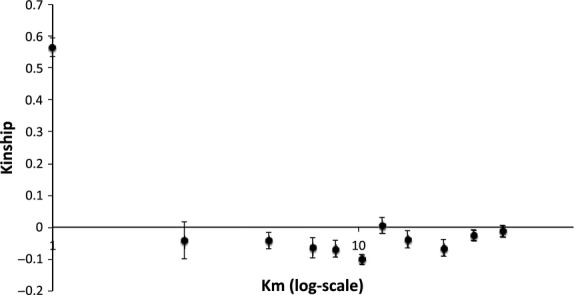
Kinship plotted against distance with standard error bars. The first point represents within-infestation kinship, the following 10 points represent geographical distance intervals, chosen in Spagedi such that each interval contains an equal number of comparisons.

In total, 63 individuals from 13 infestations across London were genotyped at 21 loci. Due to the low sample sizes per infestation (three to seven individuals), HWE could not be rejected. Within infestations, effective allele numbers ranged from 1.28 to 2.10 across all loci (Table 3) and were therefore comparable to more thoroughly sampled infestations (see above and Booth *et al*. 2012; Saenz *et al*. 2012). Within infestations, mean kinship was high (0.566 ± 0.030), and across infestations, *F*_IS_ was 0.084 (95% CI = −0.052 to 0.226). The global *F*_ST_ value across all 13 infestations showed significant population differentiation (*F*_ST_ = 0.592, SE = 0.026, *P* < 0.001). However, we found no significant pattern of isolation by distance (Fig. 2, slope = 0.013 ± 0.016). DAPC analysis further supported high differentiation between infestations with a defined genetic cluster for each infestation (Fig. S3, Supporting information).

**Table 3:** Indices of genetic diversity of 13 infestations from across London, UK. Total is the value obtained when individuals from all localities were pooled together

	Coordinates						
Infestation	Lat	Long	*n*	*E*_A_	Allelic richness* (± SE, n=20)	*H*_E_	*H*_o_
a	51.4924	−0.2294	3	1.35	1.41 (0.12)	0.209	0.270
b	51.4693	−0.1138	3	1.62	1.65 (0.11)	0.384	0.333
c	51.5293	−0.0218	3	1.28	1.37 (0.10)	0.197	0.270
d	51.4924	−0.1674	7	1.37	1.41 (0.10)	0.223	0.211
e	51.5851	−0.2602	4	1.28	1.37 (0.10)	0.189	0.163
f	51.5333	−0.1681	7	1.54	1.59 (0.11)	0.303	0.213
g	51.4491	−0.1215	5	2.10	2.07 (0.13)	0.510	0.279
h	51.6905	−0.0338	4	1.51	1.58 (0.12)	0.287	0.298
i	51.5840	−0.1171	5	1.62	1.67 (0.09)	0.370	0.412
j	51.4799	−0.0296	4	1.49	1.55 (0.10)	0.308	0.345
k	51.3843	−0.4207	6	1.41	1.41 (0.10)	0.242	0.216
l	51.5113	−0.2679	6	1.42	1.43 (0.11)	0.228	0.213
m	51.5561	−0.1739	6	1.46	1.56 (0.11)	0.275	0.317
Total			63	3.26	2.53 (0.06)	0.680	0.266

One locus was omitted as for one infestation no individuals amplified for that locus.

*E*_A_, Effective number of alleles; *n*, Number of individuals; *H*_E_, Expected heterozygosity; *H*_o_, Observed heterozygosity.

* Sample sizes standardized to the smallest number of individuals for a locus.

### Approximate Bayesian computation analysis

A PCA performed on 100 000 randomly chosen simulated data sets showed that our propagule pool model (scenario two) produced data sets that closely matched the observed data (Fig. S4, Supporting information). The model comparison gave very strong support to scenario two. In fact, the closest 1% of data sets were all generated by scenario two. We therefore had to use 2% of simulated data sets to perform the logistic regression, which again gave almost complete support to scenario two [propagule pool model: PP 0.9998, 95% highest posterior density (HPD95): 0.9987, 1.0000] over scenario one (migrant pool model: 0.0002, HPD95: 0.0000, 0.0013). FDR was low (5.2%), indicating the robustness of our model selection. Although we had strong biological reasons to limit the upper bound of t2 to 10, its posterior distribution under scenario one suggested a higher value (Fig. S6, Supporting information) making our model comparison less trustworthy. To check its influence, we relaxed this prior for both models and ran another model choice procedure. Even though results were less clear-cut, the new analysis still favoured scenario two over scenario one (PP 0.66, FDR 8.2%). We therefore made subsequent analyses using scenario two with its original, constrained prior ranges. Posterior predictive simulations showed this scenario provided simulated data sets close to the observed data (Fig. S5, Supporting information). The deviation on the 2nd axis can be explained by the deviation of Garza-Williamson's M test statistics (significant deviations are shown in Table S5, Supporting information).

**Figure 3:**
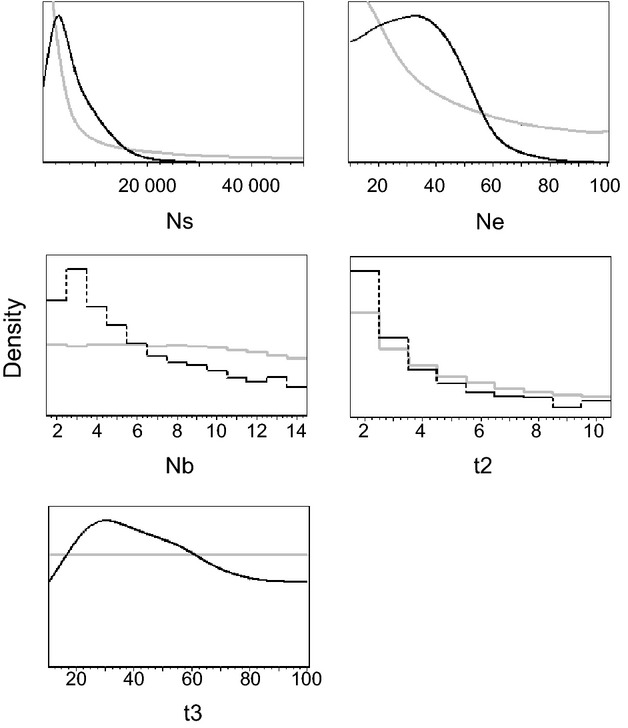
Prior (Grey) and Posterior (Black) distributions of parameters obtained under the better-supported model (scenario two – propagule pool model). The *x*-axis shows the range of parameter values, and the *y*-axis the probability density.

With a posterior modal estimation of 3, the number of founders (Nb) starting each new infestation seems to be very low (Median = 5.27, HPD90 = 2.09, 13.1; see Table 1). There was limited information regarding effective sizes of current and source populations, as well as for the divergence time from the source population (Ne, Ns and t3, respectively, see Fig. 3 and Table 1). The time since founding (t2) was low, suggesting that the sampled infestations were detected early (Median = 3.13, HPD90 = 1.75, 9.36). Whilst the mutation rate parameter was not well estimated, additional simulations showed that adjusting the prior on this parameter did not have a strong effect on the other parameters of interest and resulted in an estimate of mutation rate within the range of the original prior (Table S6 and Fig. S7, Supporting information).

## Discussion

Using a combination of descriptive and ABC genetic analyses, we have, for the first time, explicitly tested two competing models of the colonization process that underlies the dynamics of the bed bug metapopulation. First, we found very low within-infestation diversity and high differentiation between infestations. Second, ABC estimation favoured a propagule pool model, suggesting that founders are few and related (Whitlock & McCauley 1990). Low *F*_ST_ within infestations suggests that the infestation is the lowest level of population structure and not the refuge, which is consistent with a single founding event per infestation. Very high differentiation between infestations suggests limited connectivity (either via multiple sources of colonists or migration between extant infestations) and the lack of isolation by distance at the city scale fits our prediction of colonization via passive dispersal, which is likely to weaken the relationship between genetic and geographical distance (Johannesen & Lubin 1999; De Meester *et al*. 2002; Colson & Hughes 2004).

### Limitations and promise of our approximate Bayesian computation approach

Although theoretical studies have shown that the propagule and the migrant pool models may produce somewhat similar differentiation patterns (Wade & McCauley 1988; Pannell & Charlesworth 1999), our results show that an ABC approach based on multiple summary statistics can help to distinguish them. Using a high number of populations with ABC means, there may be a high dimensionality to the analysis, as it can inflate the number of summary statistics used. This can be problematic, as it often limits the ability to simulate data close enough to the observed data, thus leading to inaccurate and potentially biased scenario choices and parameter estimates (Beaumont *et al*. 2002; Blum *et al*. 2013). In our case, the large number of summary statistics was primarily due to the use of pairwise *F*_ST_ statistics. However, our PCA clearly shows that scenario two had the potential to simulate data that were representative of our observed data (Fig. S4, Supporting information: 90% of the variance explained by both axes). The GoF analysis (Fig. S5, Supporting information) further suggests that our choice of summary statistics, whilst surely not optimal, was still good enough to make valid inferences. It should also be noted that whilst our models are relatively simple, they are biologically relevant to this system in that they capture the key phases of colonization, growth and extinction. A more ‘realistic’ model may have come at the cost of information loss for our parameters of interest. In fact, we simulated a 3rd scenario in addition to the two presented here. This scenario was similar to model two but with an additional bottleneck after the initial divergence from the source population (i.e. a bottleneck occurring between the large panmictic population and the first local population, between times t3 and t2). Despite this being more realistic, we lost information about our demographic parameters and thus we chose to disregard this model.

Scenario two, arguably the more biologically realistic of the two scenarios, was favoured and posterior predictive simulations gave good predictions of the observed data. The deviations observed for some test statistics between their predicted distributions and their observed values are likely a result of this being a simplified model. As noted above, we did not expect to be able to obtain robust estimates of Ns and μ, which together determine global population diversity. Adjusting the mutation parameter priors had little effect on the key parameters of interest (see Supplementary Information). Within-infestation diversity was expected to depend on Ne and Nb as well as the time parameters. The lack of information for Ne is likely due to the strong bottlenecks and very fast subsequent population expansions within infestations. As there was a maximum of 10 generations between bottlenecks and sampling, there was not enough time for new mutations to arise, and so there was little constraint on the estimate of infestation size. Whilst care must be taken when using the ABC estimates for interpretation, it is important to note that, when taken in combination with the genetic analysis, our models produce biologically realistic estimates. We can also note that our study is in accordance with previous ones (e.g. Estoup *et al*. 2004) by showing that ABC is able to make reliable estimates of demographic parameters even when using multiple populations and whilst working within a very short evolutionary timescale.

### Bed bug infestation population dynamics

The very low levels of genetic diversity observed within infestations in this study suggest low numbers of colonists. These patterns are reflected in other descriptive surveys of the genetic structure of bed bug populations (Booth *et al*. 2012; Saenz *et al*. 2012). High null allele frequency estimates and departures from HWE have been found in other genetic surveys of highly structured metapopulations (Johannesen & Lubin 1999; Massonnet *et al*. 2002; Kankare *et al*. 2005; Orsini *et al*. 2008). Despite several markers having high null allele frequency estimates in this study, it is likely that these estimates are an artefact of the lower than expected heterozygosity resulting from founder events and inbreeding.

Due to living in aggregations and a high male mating rate, it is likely that most adult female bed bugs have been multiply mated (Stutt & Siva-Jothy 2001; Reinhardt *et al*. 2011). With low levels of genetic diversity, a female that has been mated over 10 times may have only ‘effectively’ mated once or twice, as she will likely only encounter related males. The ABC estimates are consistent with this, with a clear mode at Nb = 3. This modal value potentially represents a single female mated several times by genetically similar males, a version of the propagule pool model. This can explain the genetic data where high relatedness and kinship are detected within infestations. *F*_IS_ values have varied considerably across studies, but with few significant departures from zero (Booth *et al*. 2012; Saenz *et al*. 2012). This is likely a result of a low number of colonists with chance allele frequency differences between male and female founders resulting in variation in the proportion of heterozygotes relative to HWE. Despite low diversity, infestations can expand rapidly, suggesting there are limited costs to inbreeding, and this is supported by preliminary experiments performed by Johnson (1941). The apparent lack of inbreeding depression may be due to the continuous purging of deleterious alleles through repeated founder events (Hedrick 1994; Facon *et al*. 2011). Further work is needed to examine the true cost of inbreeding in bed bugs.

Whilst generally consistent patterns of low diversity have been observed within subpopulations, there is some variation in the genetic composition of infestations. For example, some studies have reported higher genetic diversity within infestations, which would be more consistent with the migrant pool model. Szalanski *et al*. (2008) reported one to six different mitochondrial haplotypes within infestations in a survey of 11 different properties. Booth *et al*. (2012) surveyed multiple apartments from residential blocks and found evidence of substructuring within two of the three buildings. In contrast, the survey by Saenz *et al*. (2012) showed that only one of 21 properties had evidence of multiple, genetically distinct introductions, therefore more closely following the predictions of the propagule pool model. This variation suggests that the chance of introductions from multiple sources is largely dependent on the type of property (i.e. single residence or multi-dwelling), with a greater turnover of humans increasing the likelihood of multiple founders from different sources.

Due to the stochastic distribution of genetic diversity generated by high levels of population turnover, large sample sizes are often required to detect patterns of isolation by distance (Giles & Goudet 1997; Massonnet *et al*. 2002; Haag *et al*. 2005). As this study was not particularly designed to test for patterns of isolation by distance, we may have lacked the resolution to detect it, and in fact some weak patterns have been found in other studies (Saenz *et al*. 2012). Further work is still needed to determine the overall level of connectivity between subpopulations and whether several large infestations are acting as sources for multiple patches or whether all infestations can be traced back to a larger mixed source population in an area where bed bug numbers have been consistently high.

### Metapopulation dynamics

Due to discontinuous habitat, temporal variation in the environment and small local population sizes, metapopulation dynamics should be fairly common in insects. However, classic metapopulations appear to be comparatively rare (Driscoll 2008; Driscoll *et al*. 2010), potentially because of low population turnover (Fronhofer *et al*. 2012). The close association between humans and bed bugs has led them to fulfil at least three of the four conditions required to be a classical metapopulation. Each separate infestation can be considered a discrete breeding patch, with bed bugs aggregating around a host food source, and infestations are likely to have similar effective population sizes. Pest control causes the independent local extinction of subpopulations, whilst human-facilitated dispersal gives the opportunity for recolonization. However, reports of bed bug population expansion suggest that colonization and extinction are not at equilibrium (Reinhardt & Siva-Jothy 2007). At present there is no precise information on the rate of population expansion (although see Richards *et al*. 2009), so the degree to which colonization is outweighing extinction is not known.

We have provided evidence that bed bugs experience extremely severe genetic bottlenecks during founder events and that all founders of a new colony most often originate from a single source population. As predicted, the likely common origin of colonists causes particularly strong differentiation (Whitlock & McCauley 1990). The relatively short lifespan of each infestation makes it unlikely that there will be an introduction of novel alleles via gene flow, thus maintaining these levels of differentiation.

Examples of species that can be considered metapopulations have been shown to exist on a continuum, ranging from migrant pool colonizers (Giles & Goudet 1997; Colson & Hughes 2004; Yang *et al*. 2008), to intermediates (Whitlock 1992; Austin *et al*. 2011) and examples with a high likelihood of a common origin of founders (Ingvarsson *et al*. 1997). The dynamics highlighted here through our ABC results demonstrate that bed bugs are an excellent model system to further investigate human impact on population structure and its implication for diversity, as well as pest control.

### Implications for control

Metapopulation frameworks have long been used to formulate efficient pest management strategies (e.g. Levins 1969; Cloarec *et al*. 1999; Booth *et al*. 2011). Here, the general low observed diversity within infestations suggests a single introduction to each infestation. Type of dwelling, however, is likely to strongly influence the chances of having multiple introductions. Hotels, apartment blocks and hospitals, which have the highest turnover of visitors, are likely to be the most at risk from multiple introductions (Doggett & Russell 2008). However, from the reported data so far, it seems these cases may, for the moment at least, be in the minority and reported repeated infestations may be the result of failure to fully eradicate existing infestations (Boase 2008). The novel microsatellite markers described here, in conjunction with those described in Booth *et al*. (2012), could be used to determine whether a repeat infestation is a result of pest management failure or is a recolonization event. This would require surveying properties before or immediately after treatment and keeping specimens for future study should a repeat infestation occur. This study also shows that due to the low diversity within and high differentiation between infestations, only a relatively small number of individuals is required to test for kinship between samples; therefore, sampling can be efficiently integrated into the control process. Another factor to consider is that bed bugs are becoming rapidly resistant to insecticides. Infestations containing resistant individuals are more likely to avoid extinction, prolonging their time as a source. Resistant alleles would be quickly selected for and become fixed in populations. The parameter estimates provided here could be used in modelling the spread of resistance, as well as the efficacy of control treatments.

## Data accessibility

The 329 microsatellite sequences isolated during the development of the genomic library including those of the 21 loci characterized in this study have been submitted to the EMBL database (HF969864–HF970194) and microsatellite genotyping data are available in DRYAD, doi:10.5061/dryad.cg10d

## Supporting information

Table S1 Within-infestation diversity sample information.

Table S2 Description of newly designed primers arranged into five multiplex panels.

Table S3 Pairwise *F*_ST_ estimated using Weir & Cockerhams's 0 (1984) between 11 refugia in the LON2 infestation.

**Table S4** Pairwise *F*_ST_ estimated using Weir & Cockerhams's 0 (1984) between five *C. lectularius* infestations. **Table S5** Posterior predictive probability (*P*) of observed statistics given scenario 1 or 2 (attached in a supplementary Excel file). **Table S6** Prior and posterior distributions of model parameters from additional simulations using diyabc v2 (Cornuet *et al.* in press, doi:10.1093/bioinformatics/btt763). **Fig. S1** A map of the sampling locations in the city of London, UK used to test between-infestation diversity. **Fig. S2** Ordination plot of DAPC genetic clusters in the within-infestation data set, including a minimum-spanning tree based on the squared distances between infestations within the entire space. **Fig. S3** Ordination plot of DAPC genetic clusters in the metapopulation diversity data set, including a minimum-spanning tree based on the squared distances between infestations within the entire space. **Fig. S4** Principal components analysis of summary statistics of 100 000 simulated data sets generated with two demographic scenarios (red = scenario one, green = scenario two). **Fig. S5** Principal components analysis of summary statistics from 500 pseudo-observed data sets generated from the posterior predictive distribution of scenario two (large green dots), plotted along with summary statistics of simulations generated from priors of scenario one and two (small red and green dots respectively). **Fig. S6** Prior (Red) and Posterior (Green) distributions of parameters obtained under the least supported scenario (scenario one). **Fig. S7** Prior (Red) and Posterior (Green) distributions of parameters obtained under scenario two using the adjusted mutation rate (Table S6, Supporting information).
