# Supplementary material for: Human-facilitated metapopulation dynamics in an emerging pest species, Cimex lectularius
Source: Mol Ecol. 2014 Feb 17;23(5):1071–84. doi: 10.1111/mec.12673 (PMC4016754; doi:10.1111/mec.12673)
Supplement: Table S1 — Within-infestation diversity sample information. [file mec0023-1071-sd1.doc]

Supporting Information for online publication only

Manuscript:

HUMAN FACILITATED METAPOPULATION DYNAMICS IN AN EMERGING PEST SPECIES, *CIMEX LECTULARIUS*

Toby Fountain, Ludovic Duvaux, Gavin Horsburgh, Klaus Reinhardt, Roger K. Butlin

*Microsatellite isolation*

Microsatellite sequences were isolated using two methods, the creation of a microsatellite-enriched library and through analysis of a recently available transcriptome assembly (Otti and Reinhardt, in prep). First, genomic DNA was extracted using the ammonium acetate precipitation method described by Nicholls *et al.* (2000). A microsatellite-enriched library was constructed using the method of Armour *et al.* (1994). The library was enriched for the following di- and tetranucleotide microsatellite motifs: (GT)n, (CT)n, (GTAA)n, (CTAA)n, (TTTC)n and (GATA)n, which had been bound to magnetic beads by following the procedure described by Glenn & Schable (2005). Transformant colonies were not screened for the presence of a repeat but were directly sequenced by the NERC Biomolecular Analysis Facility at the University of Edinburgh. For the transcriptome approach, transcripts were screened for microsatellite sequences using the program MSATcommander (Faircloth 2008).

In total 436 sequences containing unique microsatellite regions were isolated (329 from the genomic library and 107 from the transcriptome). However, the majority of the sequences contained only short flanking regions or had compound repeats, leaving only 62 sequences that were useful for primer design. Primer sets were designed from these sequences using the program PRIMER3 (Rozen & Skaletsky 2000). These primers were tested for amplification using four individual bed bugs from a lab stock over a gradient of 12 different annealing temperatures (56 – 64**°**C) using a DNA Engine 2 thermal cycler (MJ Research, Bio-Rad, Hemel Hempstead, Herts, UK). A further 24 individuals were then genotyped at the polymorphic loci using the temperature that produced the clearest and most consistent product when observed on an ABI3730 48-well capillary DNA analyser (Applied Biosystems, California). Each PCR contained approximately 10ng of genomic DNA, 0.2µM of each primer and 1µl QIAGEN multiplex PCR mix (QIAGEN Inc; (Kent*a et a*l. 2008)). PCR amplification was performed using a DNA Engine Tetrad Thermal Cycler (MJ Research, Bio-Rad, Hemel Hempstead, Herts, UK) with the following touchdown program: 95°C for 15 minutes, followed by 11 cycles of 94°C for 30 seconds, annealing temperature of 65°C (which decreases by 1°C each cycle) for 1 minute 30 seconds, and 72°C for 1 minute. Then 26 cycles of 94°C for 30 seconds, 55°C for 1 minute 30 seconds and 72°C for 1 minute, then finally 72°C for 10 minutes. Amplified product was then analysed using an ABI3730 48-well capillary DNA analyser and allele sizes assigned using GENEMAPPER v.3.7 (Applied Biosystems, California).

*Microsatellite characterisation*

From the 39 genomic-library designed primers 15 loci did not amplify and a further 8 were monomorphic or yielded no specific product and from the 20 transcriptome designed primers 10 markers failed to amplify and five were monomorphic. For the 21 remaining markers the number of alleles per locus ranged between three and 12 with an average of 7.4 alleles per locus across the 18 infestations sampled in this study (see Table 4). All markers consistently amplified and yielded unambiguous product suitable for analysis. Null allele estimates were high (>0.2) for five markers but this may be an artefact of general low observed heterozygosity across loci caused by recent severe bottlenecks in the sampled infestations (see ‘*Within infestation diversity’* in the main text). No marker was found to be sex linked and after Bonferroni correction no significant linkage disequilibrium was detected between markers.

**References**

Armour JA, Neumann R, Gobert S, Jeffreys AJ (1994) Isolation of human simple repeat loci by hybridisation selection. *Hum. Mol. Genet.* **3**(4), 599-605.

Cornuet J-M, Pudlo P, Veyssier J, Dehne-Garcia A, Gautier M, Leblois R, Marin J-M, Estoup A (accepted in Bioinformatics) DIYABC v2.0: a software to make Approximate Bayesian Computation inferences about population history using Single Nucleotide Polymorphism, DNA sequence and microsatellite data.

Faircloth BC (2008) MSATCOMMANDER: detection of microsatellite repeat arrays and automated, locus specific primer design. *Molecular Ecology Resources*, **8**(1), 92-94.

Glenn TC , Schable NA (2005) Isolating microsatellite DNA loci. *Meth. Enzymol.* **395**, 202-222.

Kenta T, Gratten J, Haig NS *et al.* (2008) Multiplex SNP-SCALE: a cost-effective medium-throughput single nucleotide polymorphism genotyping method. *Molecular Ecology Resources*, **8**, 1230-1238.

Nicholls J, Double M, Rowell D, Magrath R (2000) The evolution of cooperative and pair breeding in thornbills Acanthiza (Pardalotidae). *Journal of Avian Biology*, **31**, 165–176.

Rozen S, Skaletsky H (2000) Primer3 on the WWW for general users and for biologist programmers. *Methods. Mol. Biol.*, **132**, 365-386.

**Tables and Figures**

Table S1. Within infestation diversity sample information

Table S2. Description of newly designed primers arranged into 5 multiplex panels. T_a_ = annealing temperature, origin of the markers (Microsatellite library, transcriptome) and N_A_ = total number of observed alleles across 19 infestations. Markers Cle001 and Cle006 (in bold) were not used in the within-infestation analysis due to problems with an early version of the multiplexing.

Table S3. Pairwise F_ST_ estimated using Weir and Cockerhams’s *θ* (1984) between 11 refugia in the LON2 infestation

Table S4. Pairwise F_ST_ estimated using Weir and Cockerhams’s *θ* (1984) between five *C. lectularius* infestations

Table S5: Posterior predictive probability (P) of observed statistics given model 1 or 2 (attached in a supplementary Excel file). For each statistic, P is the proportion of predictive simulations showing a greater value than the observed data and is significant if P<0.025 or P>0.975.

Table S6 Prior and posterior distributions of model parameters from additional simulations using DIYABC v2 (Cornuet et al. accepted; http://www1.montpellier.inra.fr/CBGP/diyabc/). DIYABC v2 allowed the extension of the prior on mutation rate to 1 x 10^-6^ – 1 x 10^-3^. Priors for all other parameters remained the same as in the original analysis. 1 x 10^6^ simulations were performed for each scenario. The parameter estimates for scenario two are shown.

Figure S1: A map of the sampling locations in the city of London, UK used to test between-infestation diversity. Each region corresponds to a postcode area and each letter an individual infestation. Infestations h and k are located outside the city and at co-ordinates 51.6905N, -0.0338W and 51.3843N, -0.4207W, respectively.

*
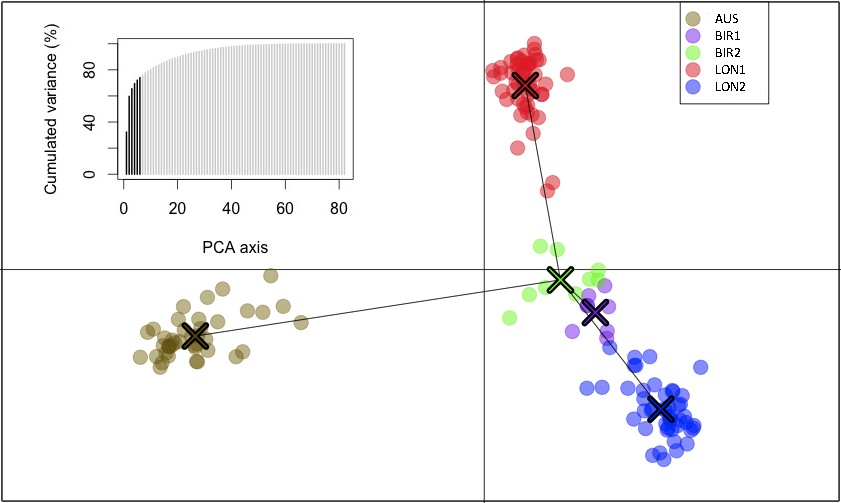
*

Figure S2 Ordination plot of DAPC genetic clusters in the within-infestation dataset, including a minimum spanning tree based on the squared distances between infestations within the entire space. Centre of each cluster is designated with an X. Cumulative variance plot shows the number of retained principal components in the analysis. All discriminant functions were retained.

Figure S3 Ordination plot of DAPC genetic clusters in the metapopulation diversity data set, including a minimum spanning tree based on the squared distances between infestations within the entire space. Centre of each cluster is designated with an X.


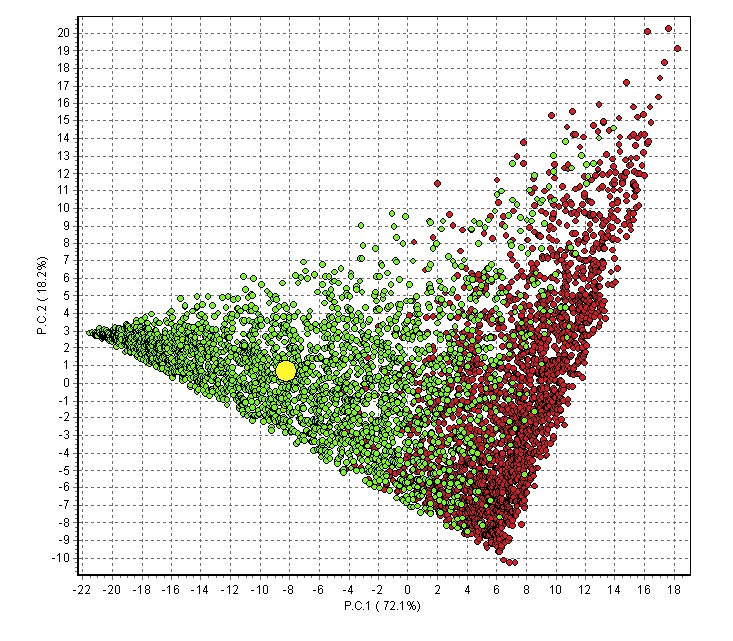


Figure S4 Principal components analysis of summary statistics of 100,000 simulated data sets generated with two demographic scenarios (red = scenario one, green = scenario two). Observed dataset (yellow dot) is projected on the plane formed by the first two principal components.


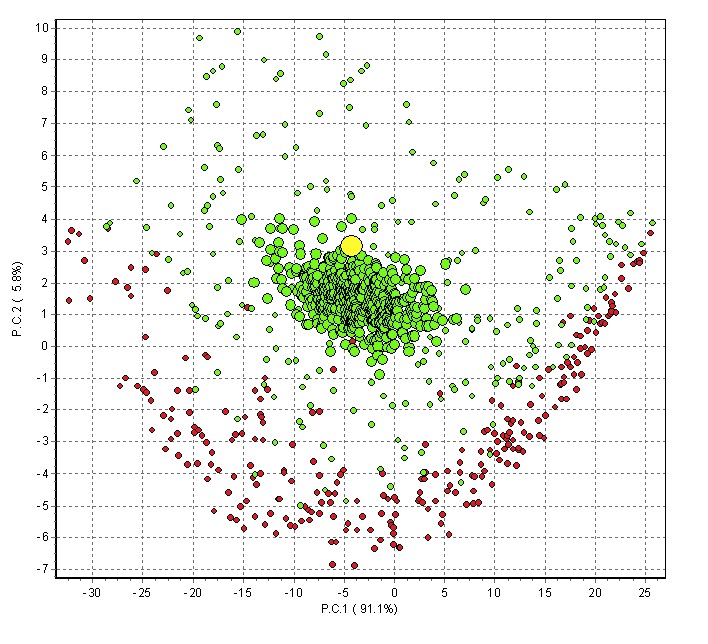


Figure S5 Principal components analysis of summary statistics from 500 pseudo-observed datasets generated from the posterior predictive distribution of scenario two (large green dots), plotted along with summary statistics of simulations generated from priors of scenario one and two (small red and green dots respectively). The observed dataset (yellow dot) is projected on the plane formed by the first two principal components.

Figure S6 Prior (Red) and Posterior (Green) distributions of parameters obtained under the least supported scenario (scenario one). The x-axis shows the range of parameter values, and the y-axis the probability density (attached as a supplementary PDF).

Figure S7 Prior (Red) and Posterior (Green) distributions of parameters obtained under scenario two using the adjusted mutation rate (Table S6). The x-axis shows the range of parameter values, and the y-axis the probability density (attached as a supplementary PDF).
