# Supplementary figures and images for: Human-facilitated metapopulation dynamics in an emerging pest species, Cimex lectularius
Source: Mol Ecol. 2014 Feb 17;23(5):1071–84. doi: 10.1111/mec.12673 (PMC4016754; doi:10.1111/mec.12673)

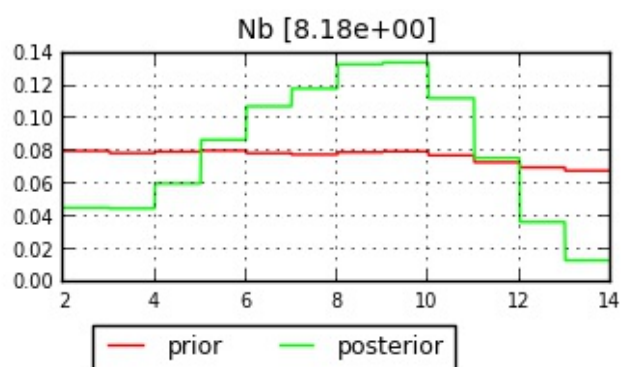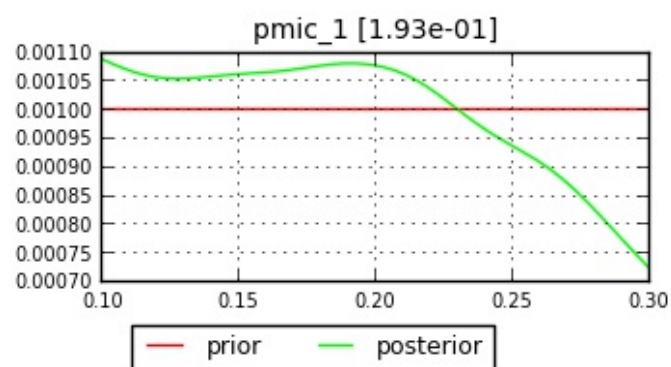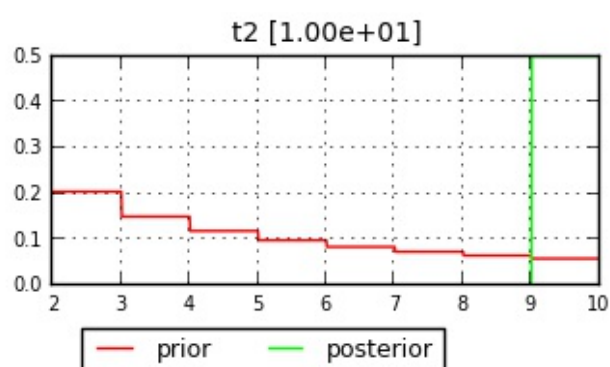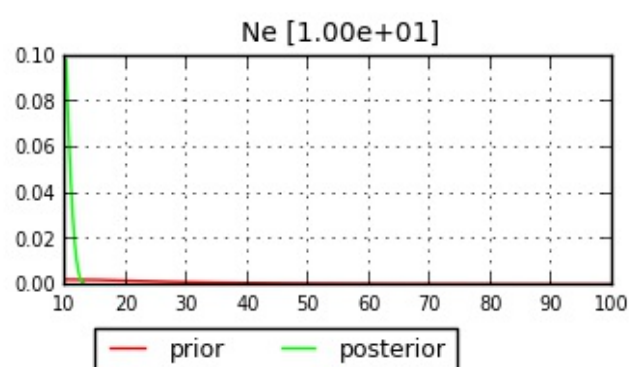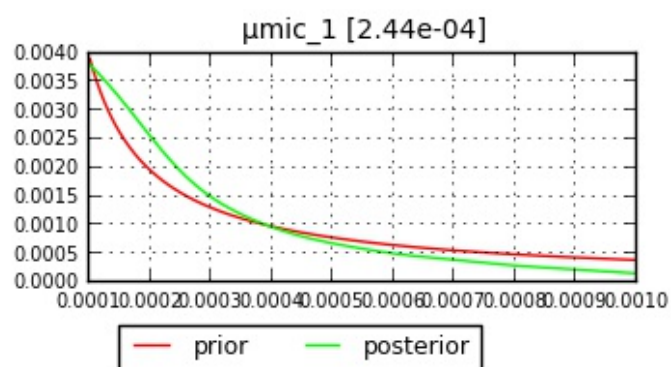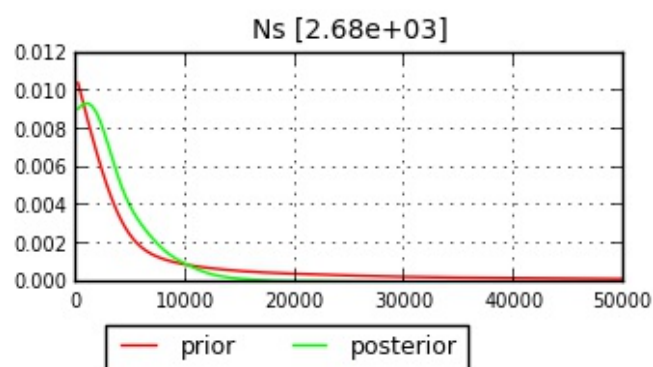

Supplement: Table S2 — Description of newly designed primers arranged into five multiplex panels. [file mec0023-1071-sd2.pdf]

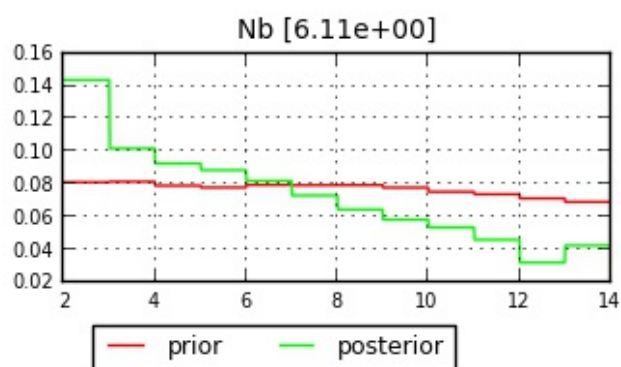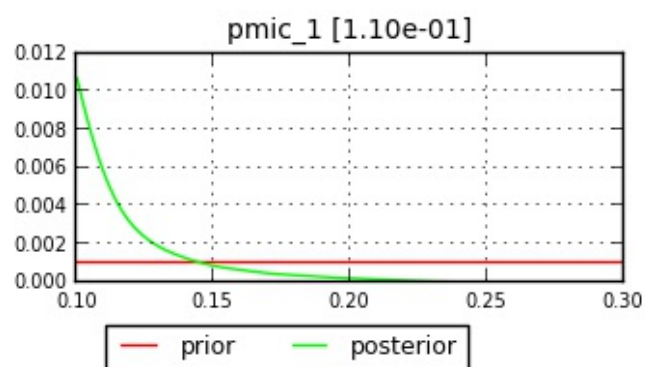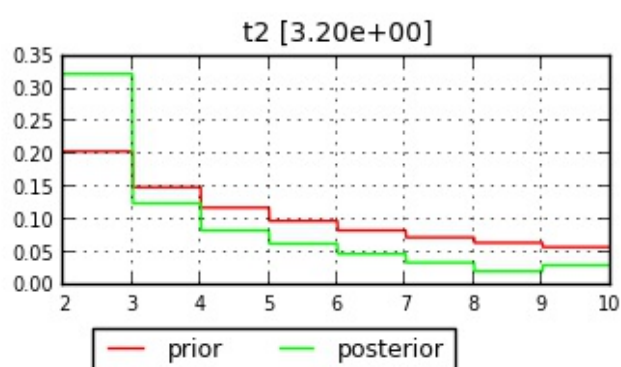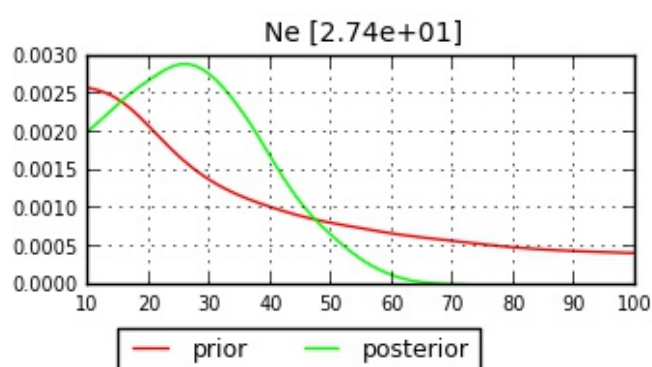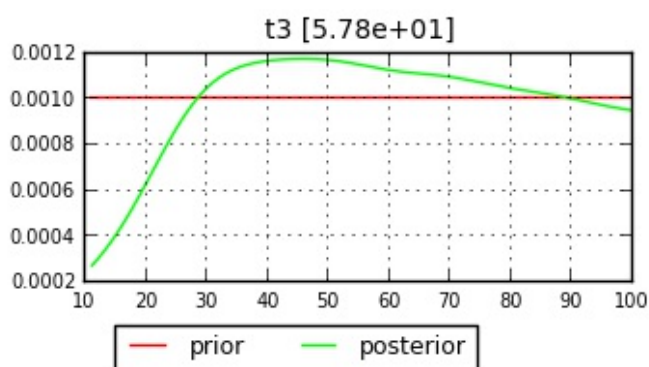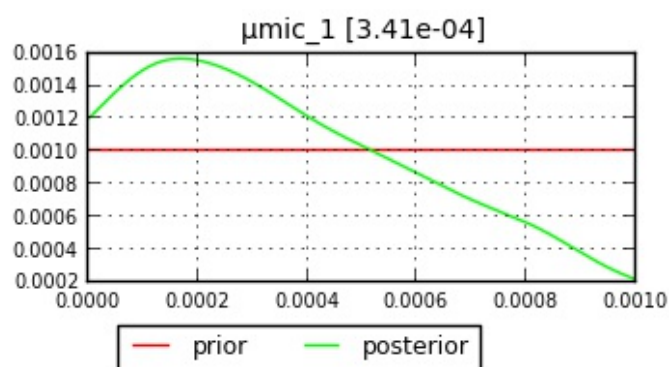

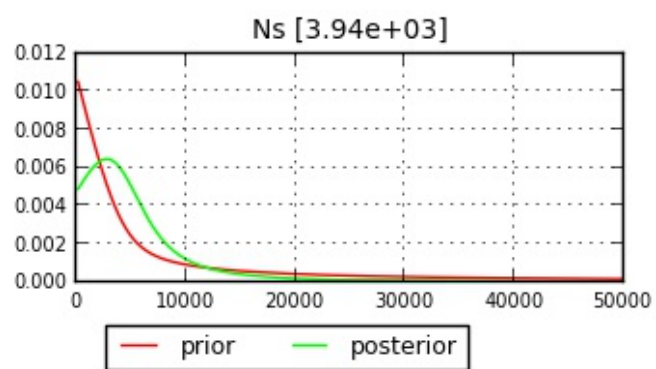

Supplement: Table S3 — Pairwise FST estimated using Weir & Cockerhams's 0 (1984) between 11 refugia in the LON2 infestation. [file mec0023-1071-sd3.pdf]
